# Carbon Nanomaterial Based Biosensors for Non-Invasive Detection of Cancer and Disease Biomarkers for Clinical Diagnosis

**DOI:** 10.3390/s17081919

**Published:** 2017-08-20

**Authors:** Tibor Pasinszki, Melinda Krebsz, Thanh Tran Tung, Dusan Losic

**Affiliations:** 1 Institute of Chemistry, ELTE Eötvös Loránd University, Pázmány Péter sétány 1/A, H-1117 Budapest, Hungary; pasinszki@chem.elte.hu; 2 School of Chemical Engineering, The University of Adelaide, North Terrace, Adelaide, SA 5005, Australia; melinda.krebsz@gmail.com (M.K.); tran.tung@adelaide.edu.au (T.T.T.); 3 ARC Research Hub for Graphene Enabled Industry Transformation, The University of Adelaide, North Terrace, Adelaide, SA 5005, Australia

**Keywords:** biosensors, biomarker, cancer detection, graphene, carbon nanotubes, graphene oxide, quantum-dot

## Abstract

The early diagnosis of diseases, e.g., Parkinson’s and Alzheimer’s disease, diabetes, and various types of cancer, and monitoring the response of patients to the therapy plays a critical role in clinical treatment; therefore, there is an intensive research for the determination of many clinical analytes. In order to achieve point-of-care sensing in clinical practice, sensitive, selective, cost-effective, simple, reliable, and rapid analytical methods are required. Biosensors have become essential tools in biomarker sensing, in which electrode material and architecture play critical roles in achieving sensitive and stable detection. Carbon nanomaterials in the form of particle/dots, tube/wires, and sheets have recently become indispensable elements of biosensor platforms due to their excellent mechanical, electronic, and optical properties. This review summarizes developments in this lucrative field by presenting major biosensor types and variability of sensor platforms in biomedical applications.

## 1. Introduction

Diseases present continuous danger to human life and well-being, and cancer is one of the leading causes of human death. The early detection and precise diagnosis of the onset of a disease is the most promising approach to accelerate healing processes or to improve survival of patients [1,2,3]. Clinical treatment and monitoring of disease recurrence after treatment also require continuous screening. To this end, fast, selective, reliable, and cheap analytical methods are required, which can be easily and routinely used even by unprofessional personnel. The human body responds to infections and development of diseases variously by producing and/or changing or fluctuating levels of certain biomolecules in the human body, and these biomarkers can be used for early detection and monitoring of diseases. Disease biomarkers belong to an important group of materials whose concentration in serum and tissue changes during the onset of a disease [1]. Taking samples from body fluids, namely urine, blood, saliva, tears, or sweat, is relatively easy, non- or minimally invasive, and provide possibility for frequent sampling thus continuous monitoring. The detection of biomarkers in body fluids, however, is challenging, because their concentration is often very low and they present in complex biological assay. Traditional assay methods such as enzyme-linked immunosorbent assay [4], radioimmunoassay [5], electrophoretic immunoassay [6], mass spectrometric immunoassay [7], and immunofluorimetric immune-polymerase chain reaction (PCR) assay [8] have some disadvantages, namely time-consuming measurement, hazardous to health, and require highly trained operators or expensive and sophisticated instrumentation. Therefore, the development of rapid, simple, and sensitive immunoassay methods for body fluid biomarker detection has a great clinical significance in the diagnosis of various diseases. Electrochemical and optical biosensors are particularly attractive for biomarker detection due to their high sensitivity, relatively easy fabricating and operating procedures, and the potential to be miniaturized. Carbon nanomaterials offer attractive opportunities for improving sensor performances due to their excellent electric and mechanical properties, high specific surface area, and biocompatibility. The incorporation of carbon nanomaterials into biosensor platforms is now a rapidly growing area of biosensor design. The most widely used carbon nanomaterials to date are nanotubes (single-walled (SWCNT) and multiple-walled (MWCNT)) and graphenes (graphene (GR), graphene oxide (GO), and reduced graphene oxide (rGO)), but carbon quantum-dots (QD) emerges as novel materials for sensor construction. In this review, we focus on the merits of carbon nanomaterials for fabrication of biosensor devices that are used as analytical tools for biomarkers detection. The analysis of multiple biomarkers, those associated with cancers or diseases, are of vital importance for early diagnosis of diseases and clinical therapy. The review also outlines some limitations and drawbacks of these developments considering practical application of nanocarbon-based biosensors for point-of-care diagnostics.

## 2. Construction of Biosensors for Biomarkers Detection

Biosensor is an analytical tool consisting of biologically active material used in close conjunction with a device that convert a biochemical signal into quantifiable signal. A typical biosensor configuration has three-element system: a bioreceptor that is responsible for selectivity of the device (e.g., enzymes, antibodies, lipid), a transducer that translates the physical or chemical change by recognizing the anatyle, and a signal-processing unit (signal output). Due to the often extremely low biomarker concentration and disease selective detection, sensitivity and selectivity are of vital importance. Other requirements include repeatability, stability, cost-effectiveness, reusability, and disposability. Common transducing elements incorporated in the biomarker biosensor platform thus far include electrochemical, optical, or mass-sensitive elements which can generate measurable current, light, or frequency signals. Overwhelming majority, ca. 89%, of biomarker biosensors developed to date are electrochemical sensors, 9% are fluorescence sensors and 2% are piezoelectric sensors (see Table 1, Table 2, Table 3, Table 4 and Table 5 below). When the analyte interacts with bioreceptor, a quantifiable signal is generated and that can be monitored by using various sensing techniques [9,10,11,12,13].

### 2.1. Electrochemical Biosensors

**Figure 1:**
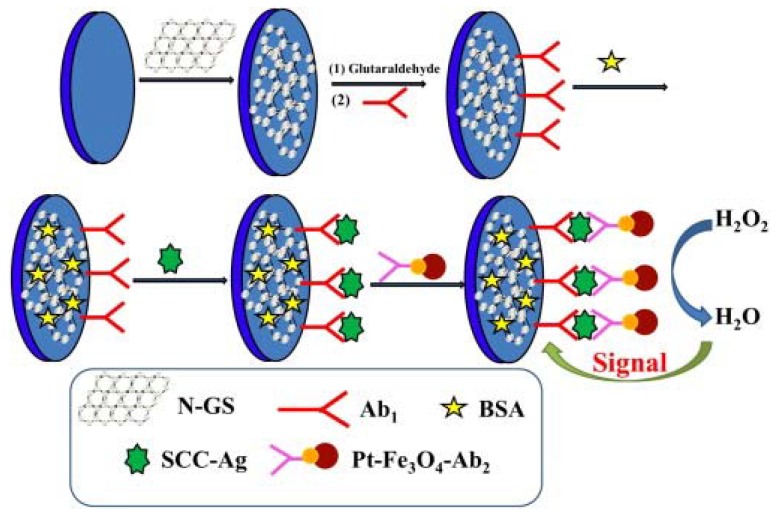
Fabrication steps of the working electrode of the sandwich-type squamous cell carcinoma antigen (SCC-Ag) biosensor by modifying a glassy carbon electrode (Ag = antigen, Ab = antibody, N-GS = nitrogen-doped graphene sheet, BSA = bovine serum albumin) [22]. Copyright 2013. Reproduced with permission from Elsevier.

These biosensors are based on electrochemical techniques in which analyte sensing is made by measuring the electrical response as an analyte reacts electrochemically with the surface of the working electrode of the sensor [14]. In general, a linear relationship between the analyte concentration and sensor response is required for practical applications. If the analyte is electroactive, e.g., glucose and dopamine, it may be detected directly with or without using a catalyst on the electrode surface. If electroactivity is insufficient or selectivity requires, the detection of the target is possible by selecting an electroactive species (e.g., hexacyanoferrate, hydrogen peroxide, etc.) and capturing the target onto the electrode surface where it acts as an inert electron and mass transfer blocking layer and hinders the diffusion of electroactive species toward the electrode surface. The sensitivity of the electrode can be increased by selectively attaching labels to the captured target (sandwich-type arrangement), which catalyse the redox reaction of the electroactive species (see Figure 1). Basic questions concerning sensitivity and selectivity are, respectively, how to increase electron transfer toward the electrode and how to make the electrode reaction specific to a selected target. In order to increase electrode response magnitude, increasing the electrode surface, incorporating good conducting materials into the electrode modifying thin film layer, and/or catalysing the redox reaction are frequently applied. Although, in general, larger surface area results in increased current response of a target due to increased number of reactive sites, increased current is offset by an increased background current which limits the sensitivity [15]. Limit of detection (LOD), usually defined as the lowest analyte concentration where the signal is three times larger than the noise, is more useful for characterizing sensor performance than sensitivity in terms of applicability. The selectivity of the sensor is a key issue, and anchoring selective target capture target antibodies on the electrode surface is generally applied. This is the method of choice for cancer sensors where the concentration dependent cancer antigen detection is the major task. The formation of antibody-antigen complex in these sensors decreases the peak current of the redox pair label linearly with increasing antigen concentration. A basic DNA biosensor is usually designed by the immobilization of single stranded oligonucleotide on sensing substrates to recognize its complementary (target) DNA sequence via hybridization. The hybridization event is then converted into a readable signal by the transducers. After immobilizing target capture biomolecules or molecules on the sensor surface, it is necessary to block remaining binding sites on the surface in order to prevent nonspecific binding of the target. Depending on the surface property and binding strategy, bovine serum albumin (BSA), polyoxyethylenesorbitan monolaurate (Tween 20), and thiolated molecules on gold surfaces are widely used. Carbon nanoparticles (NP) possess excellent electrical properties and large specific surface area thus they are ideal components of biosensor platforms. There are well-established methods for their synthesis for sensor applications [15,16,17,18]. These are based on either building up nanoparticles from small molecular building blocks formed by the decomposition of a precursor (bottom-up methods, namely chemical vapour deposition (CVD) and wet chemical synthesis) or splitting bulky and layered compounds into single-layer nanomaterials (top-down methods, namely chemical, liquid, or mechanical exfoliation). Pristine carbon nanoparticles, in general, are chemically inert, and it is necessary to activate their surface for functionalization and for facilitating composite formation and immobilizing biomolecules via these functional groups [19,20,21]. Functionalization, however, may introduce a large number of defects, what reduce the electrical conductivity and corrosion resistance of nanoparticles. Extensive reduction of the carbon surface can lead to increased background current and reduced LOD [14,15]. An alternative way of functionalization is the noncovalent functionalization of carbon NPs, e.g., with metal or metal oxide NPs, biomolecules, biopolymers, and organic polymers. These materials cause little or no structural damage to carbon NPs, and composites obtained promotes electron transfer due to synergistic effects and introduce a large amount of binding sites for capturing biomolecules. Gold NPs, for example, are frequently used in biosensor construction because AuNPs are excellent electric conducting materials and it is relatively easy to immobilize thiolated molecules on their surface by chemisorptions. Carbon nanomaterials are generally used (i) in the recognition element of the sensor, where they provide binding sites for target biomarkers or molecules capturing target biomarkers, (ii) in the transducer component that converts the detected molecular interaction on the electrode surface into a measurable signal, and (iii) labels for target biomarkers in signal amplification. The biosensor performance strongly depends on these components, and can be tuned by modifying them. In general, the LOD can be increased if less noise is observed compared to the current in the working electrode upon analyte interaction. 

Immobilizing target capture antibodies on the electrode surface plays an important role in biosensor construction. Large fraction of the known biomarker antibodies, e.g., proteins, has amine groups, and these can be immobilized covalently by using these amine residues and carboxyl functional groups of carboxylated carbon NPs or modifiers on the sensor surface. Molecules containing both the carboxyl and thiol groups can be used as linkers to functionalize gold and silver nanoparticles. Carboxyl group, however, has to be activated for this reaction. The most widely applied activation method is based on the application of 1-ethyl-3-(3-dimethylaminopropyl)-carbodiimide (EDC) and N-hydroxysuccinimide (NHS) (EDC/NHS coupling, see e.g., [23]). Amino-groups of capture antibodies and amino-functionalized carbon NP composites, e.g., amino-groups of chitosan, are usually linked using glutaraldehyde (GLA coupling, see e.g., [24]). Other less frequently used methods to immobilize biomarker capture antibodies utilizes biospecific lectin-sugarprotein interaction [25], biotin-streptavidin interaction [26], and bonding between amino groups and Pt [22].

### 2.2. Optical-Based Biosensors

**Figure 2:**
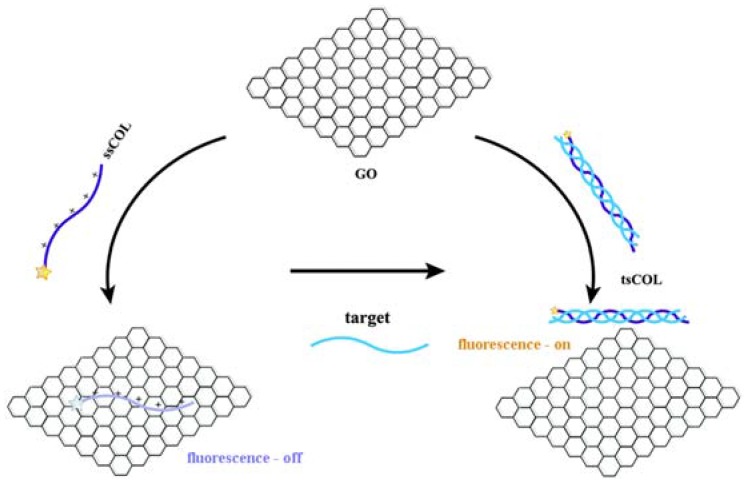
Illustration of the working principle of a GO-based fluorescence sensor utilizing the hybridization of single stranded collagen (ssCOL) peptide with complementary target collagen sequences to form triple stranded collagen (tsCOL) [28]. Copyright 2015. Reproduced with permission from Royal Society of Chemistry.

These sensors are based on detecting changes in the emission of light upon target-recognition element interaction. Carbon NPs, especially graphene derivatives, are efficient fluorescence quenchers [9]. The solubility of GO and rGO, and their functionalized derivatives in water provides possibility for their application in aqueous environments, namely in biological samples. A GO or rGO based fluorescence sensor, in general, is based on a fluorophore covalently attached to a target capture probe, what is capable to adsorb non-covalently to GO, but released from the GO surface upon binding to the target. Adsorption leads to quenching the fluorescence, but it is restored upon target-capture probe interaction. The most typical example is a labelled single stranded DNA (ssDNA) capture probe complementary to target ssDNA; the capture ssDNA adsorbs to GO, but the double stranded dsDNA desorbs from the GO surface after target-capture probe hybridization. This fluorescence method is attractive for bioassay application, because it is simple, the signal intensity is high, the background noise is low, and it is able to realize multiple target and multicolour detection [27]. Although there are several variants of this method [27], most of these sensors thus far are based on ssDNA capture antibodies. The application of optical sensors for disease and cancer biomarker detection is relatively rare to date, which may be explained by the fact that the majority of the currently known cancer and disease biomarkers are not DNA derivatives. Inspired by GO-based DNA biosensors, a GO platform for sensing collagen triple helix was developed recently [28]. The biosensor is based on dye-labelled single stranded collagen (ssCOL) peptide probe, what adsorbs to GO and targets its complementary collagen peptide. The working principle, identical to DNA sensors, is shown in Figure 2.

### 2.3. Piezoelectric-Based Biosensors

Sensor devices are based on piezoelectric crystals, mainly quartz that vibrates under the influence of an electric field [11]. Since the mass of the adsorbed material to the crystal is proportional to the resonance frequency of the crystal, the concentration dependent binding of biomarkers can be monitored. The construction of quartz crystal microbalance (QCM) biosensor platform, in general, involves the fabrication of a homogeneous biocompatible film on the QCM surface and the immobilization of target capture antibodies on this surface. The piezoelectric immunosensors are reported to be one of the most sensitive devices developed to date, being capable of detecting antigens in the picogram range. Moreover, this type of device is believed to have the potential to detect antigens in the gas phase as well as in the liquid phase.

Although QCM biosensors possess high sensitivity, real-time output, label- or radiation-free entities, and simplicity, they are hardly applied for carbon NP based biomarker sensor constructions to date, possibly due to the difficulty in obtaining a homogeneous biocompatible film with good adhesion abilities to transducer components and relatively high cost [29]. In conclusion, this PZ biosensors still need considerable development before they can be considered to become a routine anatical tool.

## 3. Detection of Cancer and Disease Biomarkers

Biomarkers present in body fluids include a wide variety of materials such as small molecules (e.g., glucose, dopamine, and uric acid), RNAs, peptides, proteins, DNAs, polysaccharides, and lipids. The detection of antigens is especially important for cancer diagnosis. Carbon nanomaterials offer novel routes to design new biosensors due to their unique properties. Functionalization or hybridization of these materials with biopolymers, organic polymers, metal oxide nanoparticles (NP), and metal NPs expand application possibilities due to synergistic effects and introduce a large amount of binding sites for capturing biomolecules or immobilizing biomarker capture antibodies. Most widely used biomarker capture probes are ssDNAs, e.g., aptamers, and proteins, e.g., antigen antibodies. Immunoassays based on the antibody-antigen, aptamer-RNA, aptamer-protein, and protein-protein interaction are the most important analytical methods for the quantitative detection of biomarkers. 

### 3.1. CNT-Based Biomarker Biosensors

CNTs promote electron transfer and possess high stability, low background noise, rapid electrode kinetics, and excellent biocompatibility. Therefore, CNTs are widely used in various biosensor constructions for preparing the sensing layer of the sensor and for fabricating labels for signal amplification in sandwich-type biosensors [19,30,31,32].

#### 3.1.1. CNT-Based Biosensors for Cancer Biomarker Detection

Prostate-specific antigen (PSA) is a single chain glycol-protein, and it is the most widely used biomarker for prostate cancer. Several CNT-based biosensors were constructed for PSA detection using anti-PSA antibodies as recognition elements on the sensor surface (Table 1). These sensor platforms include microelectrode arrays modified with SWCNTs [33], cross linked starch functionalized MWCNT-gold NP nanocomposite film [34], SWCNT forest-primary antibody platform with multi-label secondary antibody-MWCNT-horseradish peroxidase (HRP) bioconjugate labels [35], and gold NPs functionalized polypyrrole (PPy)@MWCNT nanocomposite with HRP-conjugated anti-PSA labels [36]. The LOD of sensors gradually decreased to 1 pg/mL by incorporating AuNPs and polymers into the sensor platform and applying target labels. Osteopontin (OPN), a phosphoprotein, is also a prostate cancer biomarker. OPN immunosensors were fabricated by Lerner et al. [37] and Sharma et al. [38] by covalently attaching monoclonal anti-OPN antibodies to CNTs of a field-effect transistor (FET) and a transparent chemiresistor-type sensor, respectively. These sensors exhibited an antigen-specific, concentration dependent response, wide linear range, and high sensitivity (Table 1). MicroRNAs (miRNAs), small non-protein-coding ss-RNA molecules consisting of 18 to 30 nucleotides, are biomarkers for various human cancers. miRNA-21 and miRNA-141, for example, are prostate cancer biomarkers. An ultrasensitive miRNA-21 biosensor based on layer-by-layer assembly of SWCNT, nanodiamonds (NDI), SWCNT, and AuNP on gold electrode was constructed by Liu et al. [39]. Tetrahedron-structured probe (TSP) capture DNAs were immobilized on the AuNPs through Au-S bonds in order to capture miRNAs by hybridization. The signal was amplified by DNA functionalized AuNPs modified with long hemin-G-quadruplex DNAzyme nanowires. Two miRNA-141 biosensors were constructed by Tran et al. [40,41] based on amino-modified 22-mer DNAs as miRNA capture probes. The label-free sensor [40] fabricated by co-electrooxidation of 5-hydroxy-1,4-naphthoquinone (JUG) and 3-(5-Hydroxy-1,4-dioxo-1,4-dihydronaphthalen-2(3)-yl) propanoic acid (JUGA) monomers on MWCNT modified glassy carbon electrode (GCE), and the sandwich-type electrode [41], based on MWCNT-rGO composite modified gold screen-printed electrode (GSCE) and HRP-conjugated secondary antibody labels, exhibited comparable LOD and linearity range (Table 1). A sensitive and selective biosensor for the detection of miRNA-122a was constructed by Ramnani et al. [42] by integrating an extremely sensitive SWCNT-FET transducer and a highly selective biorecognition element of Carnation Italian ringspot virus p19 binding protein. Tian et al. [43] developed a lung-cancer related let-7 miRNA sensor based on CNT enhanced label-free detection and hairpin (HP) DNA probe triggered solid-phase rolling-circle amplification (RCA). The sensor exhibited ultrasensitive detection limit and excellent specificity for let-7 miRNA. Li et al. [44] constructed a sensitive miRNA-24 sensor by drop-casting MWCNTs on GCE and immobilizing aminated capture probe ss-DNAs on the electrode surface. 

**Figure 3:**
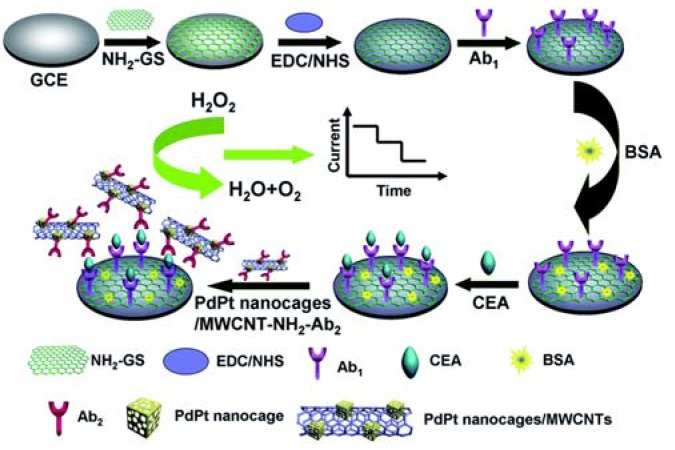
Illustration of the fabrication steps and working principle of the sandwich-type carcinoembryonic antigen (CEA) immunosensor (Ab = antibody, NH_2_-GS = APTES-functionalized graphene sheet, BSA = bovine serum albumin, EDC = 1-ethyl-3-(3-dimethylaminopropyl)-carbodiimide, NHS = N-hydroxysuccinimide) [54]. Copyright 2015. Reproduced with permission from Royal Society of Chemistry.

Carcinoembryonic antigen (CEA) is an important biomarker for the diagnosis of cervical carcinomas, and pancreatic, gastric, colorectal, and lung cancer. Various CNT-based electrochemical sensors were developed for CEA detection utilizing anti-CEA antibodies as target recognition elements. Sensor platforms include microelectrode arrays modified with chitosan (CS)-MWCNT-thionine (Thi) hybrid film [45], conducting paper (CP) electrode based on CNT-poly(3,4-ethylenedioxythiophene):poly(styrenesulfonate) (PEDOT:PSS) composite [46], GCE modified by multi-layer films made from Prussian Blue (PB) NPs and rGO-MWCNT composites and AuNPs [47], GCE modified by multi-layers of PB NPs and MWCNT-polyethyleneimine (PEI)-AuNP composites and chitosan with AuNPs [48], and GCE modified by layer-by-layer (LbL) assembly of positively charged CNTs wrapped by poly(diallyldimethylammonium chloride) (PDDA) and negatively charged poly(sodium-p-styrene-sulfonate) (PSS) [49]. The latter three multilayer-film modified sensors exhibited comparable sensitivity and linearity range (Table 1). Constructing relatively simple sensor surfaces by immobilizing anti-CEA antibodies directly on gold electrode via cysteine linkers [50] or on MWCNT modified SPCE [51], but using labels on targets improved sensor performances. Labels for the modified gold electrode were prepared by coating MWCNTs with PDDA and depositing HRP, ConA, and HRP-labelled anti-CEA on the nanoparticle surface. Labels for the SPCE electrode were prepared by immobilizing secondary CEA antibodies and glucose oxidase (GlOx) on gold nanorods. Applying CNT composites and metal NPs in sensor surface modification, as well as target labels further improved sensor performances (Table 1). Deng et al. [52] used PtNP dotted rGO-MWCNT composites as modifiers of the electrode surface and carbon dot (CD) functionalized Pt/Fe-NPs as nanolabels. Hu et al. [53] fabricated a photoelectrochemical biosensor based on MWCNT-Congo red (CR)-C_60_ hybrid labels and poly(*p*-aminobenzoic acid) (PABA)-MWCNT nanocomposite-modified indium-tin-oxide (ITO) electrode. Li et al. [54] developed an ultrasensitive immunosensor with LOD of 0.2 pg/mL based on PdPt nanocages/amino-functionalized MWCNTs as signal labels and APTES-functionalized graphene sheets (NH_2_-GS) as transducing materials (see Figure 3). 

Carcinoma antigen-125 (CA125) is the most frequently used clinical biomarker for ovarian cancer. Paul et al. [55] and Chen et al. [56] constructed label free CA125 biosensors by modifying GCE with MWCNTs embedded ZnO nanowire film and MWCNT-Nafion composite film incorporating tris(2,2′-bipyridyl)cobalt(III) (Co(bpy)_3_^3+^) mediator and AuNPs, respectively, as well as anti-CA125 antibodies. The GCE/MWCNT-ZnO/anti-CA125 sensor exhibited much higher sensitivity with a LOD of 0.00113 U/mL. Carbohydrate antigen 19-9 (CA 19-9) is a marker of pancreatic, colorectal, and hepatic carcinomas. Ding et al. [29] developed a piezoelectric immunoassay for CA 19-9 detection by immobilizing anti-CA 19-9 antibodies on QCM modified by poly-l-lysine/hydroxyapatite/MWCNT composite (PLL/HA/MWCNT). The tumour suppressor gene TP53 and protein p53 (AG_p53_) mutations are important early diagnostic cancer markers. A sensitive DNA biosensor was constructed by Fayazfar et al. [57] for detecting TP53 mutation. The working electrode of the sensor was prepared by synthesizing well-aligned MWCNTs on Ni-deposited Ta plate by CVD, electrodepositing AuNPs, and finally immobilizing 26-mer thiolated DNAs on AuNPs to capture target. Wang et al. [58] developed an enzyme electrocatalytic sandwich-type immunosensor for AG_p53_ detection. A GCE was modified by MWCNT-Nylon 6 (PA6)-polythionine (PTH) composite nanofibers and anti-AG_p53_ capture antibodies. The signal was amplified using HRP-conjugated secondary polyclonal anti-AG_p53_. K-ras gene mutation is highly associated with colorectal cancer. Wang et al. [59] constructed a specific K-ras bioensor by electrospinning MWCNTs doped nylon 6 (PA6) nanofibers onto a GCE, modifying the electrode surface by thionine electropolymerization, and immobilizing a 20-mer ssDNA1 to capture K-ras gene. A sandwich format of ssDNA1/K-ras gene/AuNP-20-mer ccDNA2 was prepared for signal amplification, and this latter was further increased by building a network-like structure between Au-NPs using thiocyanuric acid. High level of circulating galectin-3, a β-galactoside-binding protein, is correlated with an increased potential for malignancy. Park et al. [60] constructed a FET biosensor to detect galectin-3 using d-(+)-galactose-conjugated SWCNTs as chemical probes. The sensing platform was prepared by drop casting SWCNTs linked with d-(+)-galactose on a Mo electrode prepatterned SiO_2_ substrate, printed using conventional photolithography. 

Mucin 1 (MUC 1), a heavily *O*-glycosylated protein, is strongly expressed in the early stage of breast cancer. Chen et al. [61] developed a sandwich-type aptasensor for MUC 1 detection. The sensor was fabricated by electropolymerizing poly(*o*-phenylenediamine) (oPD) on a gold electrode, followed by Au-NP deposition and casting thiolated primary aptamers on the electrode surface. The tracing tag was prepared by depositing gold NPs, thionine, and thiolated aptamers on SiO_2_@MWCNT nanocomposites. Human epidermal growth factor receptors (HER) normally present in human adults, however, their over expression indicate risk of cancer. HER2 is an indicator of breast cancer. Arkan et al. [62] constructed a HER2 sensor by electrodepositing AuNPs onto MWCNT-carbon ionic liquid electrode (CILE), attaching carboxyl-stabilized AuNPs via 1,6-hexanedithiol (HDT) linkers, and immobilizing monoclonal anti-HER2 antibodies (Herceptin) on the electrode surface. Asav and Sezgintürk [63] constructed a highly sensitive HER-3 biosensor by immobilizing anti-HER-3 antibodies on the surface of a screen-printed carbon electrode (SPCE) modified with SWCNTs. Elevated expression of matrix metalloproteinase-3 (MMP-3) is associated with squamous cell carcinoma of the head and neck, and adrenal tumors. Munge et al. [64] constructed a MMP-3 sensor based on SWCNT forest-primary antibody sensor platform and bioconjugate labels prepared by immobilizing secondary MMP-3 antibodies and biotinylated HRP on polystyrene beds coated with streptavidin. Alpha-fetoprotein (AFP) is a biomarker of hepatocellular carcinoma, one of the most common malignant cancers. Tu et al. [65] fabricated an AFP sensor by drop casting an AuNP/CS solution on GCE and immobilizing anti-AFP on CS. SWCNT-MnO_2_ tags conjugated with AFP were deposited on the modified electrode surface for AFP detection. Yang et al. [66] constructed a label free AFP sensor by drop-coating SWCNTs on SPCE and covalently linking wheat-germ agglutinin (WGA) lectin as molecular recognition element to SWCNT. An ultrasensitive AFP sensor was developed by Li et al. [67] by applying anti-AFP recognition elements on both the sensor surface and label. The working electrode was fabricated by electrodepositing AuNPs on GCE and immobilizing AFP antibodies on the electrode surface. The signal amplification label was prepared by depositing AuNPs, lead ions, and secondary AFP antibodies on amino-functionalized MWCNT-Fe_3_O_4_. 

Simultaneous detection of several biomarkers has an elevated diagnostic value. Choudhary et al. [68] developed a biosensor for simultaneous detection of lung cancer biomarkers anti-MAGE A2 and anti-MAGE A11 using multichannel electrochemical analyser having two working graphite (GR) electrodes modified by SWCNT-CS composite in one reaction cell. Biomarker specific antigens were immobilized on the composite surface separately. Sanchez-Tirado et al. [69] used p-aminobenzoic acid functionalized double-walled carbon nanotubes (HOOC-Phe-DWCNT) to construct a sandwich-type dual electrochemical platform for the simultaneous detection of factor necrosis tumor α (TNF-α) and Interleukin 1β (IL-1β) in spiked serum and saliva. The electrode was prepared by casting HOOC-Phe-DWCNT onto screen-printed carbon electrode (SPCE) and immobilizing capture antibodies using commercial polymeric coating Mix&Co^TM^. Signal amplification was introduced by means of biotinylated antibodies and poly-HRP-streptavidin conjugates.

#### 3.1.2. CNT-Based Biosensors for Disease Biomarker Detection

Myoglobin (Mgb), Netrin 1, Myeloperoxidase (MPO), and cholesterol are important biomarkers of cardiovascular disease and myocardial infarction. Khan et al. [70] constructed a label free Mgb immunosensor by depositing monoclonal anti-Mgb antibodies onto the screen-printed-MWCNTs electrode by adsorption technique. A sensitive netrin 1 biosensor was fabricated by Xu et al. [71] by modifying GCE consecutively with MWCNT-chitosan (CS) composite film, thionine, gold NPs, and immobilizing netrin 1 antibodies on the electrode surface to capture netrin 1. A disposable electrochemical MPO biosensor was designed by Herrasti et al. [72] based on CNT/magnetic microparticles (MP) concentrated on SPCE using a magnet. In order to construct the sensor, streptavidin-coated MPs were modified with biotinylated anti-MPO antibodies, and SWCNTs were deposited onto MP surfaces for signal amplification. A label free MPO immunosensor was developed by Lu et al. [73] by immobilizing anti-MPO on GCE modified successively by DMF-MWCNTs-1-ethyl-3-methylimidazolium tetrafluoroborate (EMIMBF4) and CS-CeO_2_NP composite films. Liu et al. [74] fabricated a sensitive and disposable MPO-sensor based on modified ITO prepared by electropolymerizing a poly(*o*-phenylenediamine) (PoPD)-MWCNT-EMIMBr composite film on ITO, depositing AuNPs on the film, and immobilizing anti-MPO on AuNPs. Navamani et al. [75] prepared a sensitive cholesterol sensor by casting a film containing Nafion, MWCNT, cholesterol oxidase (ChOx), and poly-*N*-vinyl-2-pyrrolidone (PVP) encapsulated ZnS NPs onto GCE electrode. 

The specific antibody of neuromyelitis optica disease targets aquaporin-4 (AQP4), a transmembrane protein expressed in the central nerve system. Son et al. [76] constructed a CNT-FET functionalized with AQP4 extracellular loop peptides for the rapid detection of AQP4 antibody in human serum. Lyme antigens present in body fluids are key biomarkers of Lyme disease. Lerner et al. [77] developed a sensitive and rapid biosensor based on antibody-functionalized SWCNT-FET for the detection of Lyme antigens. 

α-1 antitrypsin (AAT) and Amyloid-β (Aβ) are recognised biomarkers of Alzheimer’s disease. Zhu and Lee [78] developed an aptamer-antigen-antibody sandwich-type AAT biosensor based on 3,4,9,10-perylenetetracarboxylic acid (PTCA)/CNT as sensing platform and alkaline phosphatase (ALP)-labelled AAT antibody functionalized AgNPs as signal enhancer. The working electrode of the sensor was constructed by drop casting PTCA-CNT on SPCE and immobilizing AAT specific amino-terminated 37-mer DNA aptamers, on the electrode surface. Oh et al. [79] developed a SWCNT-film based metal semiconductor FET for Aβ detection in human serum. Gold top gate was deposited on the middle of the SWCNT channel, and Aβ antibodies were immobilized on the gold layer using an antibody binding protein. Monitoring the level of acetylcholine and its precursor choline in serum is very important to detect neurodegenerative diseases such as Alzheimer’s and neuromuscular diseases. A bienzymatic choline biosensor was constructed by Pundir et al. [80] by electrodepositing MWCNT and ZrO_2_ NPs on GCE, and co-immobilizing acetylcholinesterase (AChE) and choline oxidase (ChlO) on the electrode surface. 

Human sirtuin1 (SirT1) is biomarker of age-related diseases. An et al. [81] constructed a SirT1 biosensor based on polymeric G4-polyamidoamine dendrimer (PAMAM)-Au-MWCNT nanocomposites as electrode modifiers and core-shell SiO_2_@Au NP labels. HRP-anti-SirT1 and anti-SirT1 antibodies were attached to labels and sensor surface, respectively, for sandwich-type sensing. Serum anti-citrullinated peptide antibodies (ACPAs) are specific markers for rheumatoid arthritis (RA) autoimmune disease. An electrochemical immunosensor device for the rapid detection of ACPAs in human serum was developed by de Gracia Villa et al. [82]. The immunosensor composed of MWCNT-polystyrene (PS) composite transducer and immobilized citrullinated specific peptide receptors (CSPR) specific against autoantibodies present in RA patients. Signal amplification was introduced using anti-human IgG secondary antibodies labelled with HRP. A RA-sensor based on QCM sensing was developed by Drouvalakis et al. [83]. The sensor was prepared by drop-casting a SWCNT film on QCM crystal and immobilizing CSPR on top of this film. *Clostridium difficile* toxin B (Tcd B) is one of the causative agents of antibiotic-associated diarrhea. Fang et al. [84] constructed a sandwich-type immunosensor for Tcd B biomarker detection using a multienzyme amplification strategy. The working electrode of the sensor was fabricated by LbL coating MWCNTs, Prussian blue (PB), and CS on GCE, and immobilizing primary Ted B antibodies on the electrode surface. Signal labels were prepared by immobilizing HRP conjugated Tcd B secondary antibody and HRP on GO. 

Abnormal levels of dopamine (DA), uric acid (UA), or glucose in plasma and urine are indicators of several diseases, for example Parkinson’s and Alzheimer’s disease, Lesch-Nyhan syndrome, and diabetes. Anirudhan et al. [85] constructed a molecular imprinted polymer (MIP) modified copper electrode for potentiometric detection of DA. MIP was prepared by selective polymerization of acrylamide grafted MWCNTs with itaconic acid as functional monomer in the presence of DA using ethylene glycol dimethacrylate as a cross-linker. Prasad et al. [86] fabricated a similar MWCNTs-MIP based DA sensor, but used carbon ceramic electrode (CCE) (Table 2). Ali et al. [87] fabricated a DA sensor with superior LOD of 40 pM by modifying a gold electrode with a thin layer of in situ polymerized poly(anilineboronic acid) (PABA)/ssDNA-wrapped SWCNT composite and a thin layer of Nafion film. The sensing approach combined the high permselectivity of Nafion and the high affinity of DA to boronic acid. Canevari at al. [88] constructed a DA and UA biosensor by modifying the surface of GCE with MWCNT/mesoporous silica composite film. A selective uricase (UOx)-based UA sensor was fabricated by Chen et al. [89]. The working electrode of the sensor was prepared by casting SDBS-coated SWNTs, depositing thionine, and immobilizing UOx, consecutively, on GCE surface. Wang and Musameh [90] prepared a glucose sensor based on co-immobilization of MWCNT and glucose oxidase (GlOx) within an electropolymerized polypyrrole (PPy) film on GCE. The sensor was able to measure glucose concentration in the hyper-glycemia range. Valentini et al. [91] used SWCNTs instead of MWCNTs and Au microelectrode instead of GCE for the fabrication of a similar CNT/PPy/GlOx composite film based glucose electrode. The increased sensitivity and extended linearity of this sensor (Table 2) provided possibility to measure glucose level useful also for hypo-glycemia disease. 

### 3.2. Graphene-Based Biomarker Biosensors

Graphenes (GO, rGO, GR) has unique electronic, adsorption, and fluorescence properties, thus they emerged in the last decade as powerful key elements of biosensors for detecting biomarkers [16,20,21,92,93,94,95]. Their properties can be finely tuned by controlled reduction or surface modification. Electronic conductivity, for example, is gradually increasing in the order of GO–Rgo–GR, but hydrophilicity is decreasing. The covalent functionalization of GO is especially simple due to the presence of sufficient amounts of carboxyl groups. Graphenes adsorb strongly to certain groups of biomolecules, and provide support for different targets. Graphenes quench fluorescence very efficiently, what makes them indispensable elements of optical sensors. 

#### 3.2.1. Graphene-Based Biosensors for Cancer Biomarker Detection

The messenger RNA biomarker PCA3 is related to prostate cancer. Vilela et al. [96] constructed an optical PCA3 biosensor based on NaYF4:Yb,Er upconversion NPs (UCNPs) as emitters linked to 25-mer ssDNAs as capture probes, and GO as the fluorescence quencher. UCNPs retained their fluorescence signal at the presence of target PCA3, because they did not interact with GO due to target-capture DNA hybridization. Zhang et al. [97,98] fabricated chemiresistor-type FET biosensors for prostate specific antigen (PSA) detection based on LbL self-assembled graphene composites. The multilayer was prepared by immersing the substrate into charged suspensions of poly(diallyldiamine chloride) (PDDA), poly(styrene sulfonate) (PSS), and graphene, and PSA capture antibodies were immobilized on the top graphene layer to capture the target. Sensor performances were improved [98] by suppressing flicker noise by suspending the graphene multilayer between gold electrodes of the sensor (Table 3). Another proof of the advantage of suspended structure compared to polycrystalline GR flakes was provided by Li et al. [99]. A FET biosensor based on suspended single crystalline graphene was prepared for lung cancer tumor marker (ANXA2, ENO1, and VEGF) detection. Antibodies were immobilized on graphene using poly-l-lysine. Sensor performance strongly increased due to the absence of grain boundary and substrate scattering. Li et al. [100] constructed an ANXA2, ENO1, and VEGF biosensor based on tunable graphene composites prepared by depositing PDDA, graphene, and TiO_2_ layers on a shape memory polymer using the self-assembly technique. Lung cancer biomarker capture antibodies were deposited on the surface of the composite for biomarker capture. 

**Figure 4:**
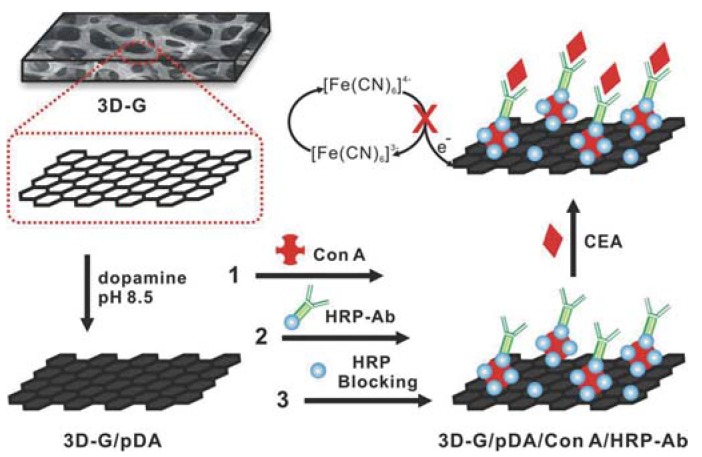
Illustration of the fabrication steps and carcinoembryonic antigen (CEA) detection process of the graphene foam electrode-based immunosensor (3D-G = three dimensional graphene, Ab = antibody, ConA = *concanavalin A*, HRP = horseradish peroxidase, pDA = polydopamine) [25]. Copyright 2014. Reproduced with permission from Elsevier.

Carcinoembryonic antigen (CEA) is elevated in many malignancies. Liu et al. [25] constructed a CEA sensor by preparing and modifying graphene foam (GF) electrode with polydopamine (pDA) linker, *concanavalin A* (conA), and HRP-labelled anti-CEA antibodies using the lectin-mediated strategy. The fabrication and detection process of the sensor is shown in Figure 4. Wen et al. [26] constructed a sandwich-type CEA biosensor based on triplex signal amplification strategy and on oligonucleotide aptamer capture probes. The sensor platform was prepared by casting a cetyltrimetylammonium bromide (CTAB)-GR suspension on GCE, followed by a chitosan (CS)-streptavidin (SA) solution. The bioconjugate labels were prepared by immobilizing thiolated and biotinylated hairpin (HP) DNA probes and HRP on gold nanorods (AuNR). At the presence of CEA, HP loops opened-up and the exposed biotins bonded to SA via avidin-biotin reaction. Multiplex sandwich-type immunosensors for simultaneous detection of CEA and α-fetoprotein (AFP) were developed by Li et al. [101], Chen et al. [102], and Wang et al. [103] by using different sensor materials and architectures, and immobilizing anti-CEA and anti-AFP antibodies on both the sensor surface and labels. Superior sensor performance was achieved by combining a polyaniline (PANI)/Au nanoparticles modified paper working electrode (Au-PWE) with 3D-rGO@methylene blue (MB) and carboxyl ferrocene (Fc-COOH) redox probe tracers [101], compared to CS-AuNPs modified GCE sensor platform with toluidine blue (TB) and Prussian blue (PB) redox probes deposited on carboxyl graphene nanosheets (CGS) [102] or ionic liquid reduced graphene oxide (IL-rGO) modified GCE sensor platform with amino capped Pt porous NPs signal tags complexed separately with Cd^2+^ and Cu^2+^ ions for antibody labelling [103]. A triple tumor marker immunosensor for simultaneous detection of CEA, PSA, and AFP was developed by Xu et al. [104]. The biosensor was based on IL-rGO and PSS modified GCE and carbon-AuNP nanocomposite labels. Primary capture antibodies were immobilized on the modified electrode surface. Biocomposite labels were prepared separately for the three target biomarkers by adsorbing thionin (Thi), 2,3-diaminophenazine (DAP), and Cd^2+^ on CAuNPs, and immobilizing anti-CEA, anti-PSA, and anti-AFP, respectively. Zhu et al. [105] constructed a sandwich-type biosensor for simultaneous detection of CEA, AFP, CA125, and PSA based on hybridization chain reaction (HCR) and biotin-streptavidin signal amplification strategy. The sensor platform was prepared by immobilizing the four antibodies simultaneously on homogenous GR-Au multilayer film modified GCE. Bioconjugate signal tags were prepared separately by linking secondary biotinylated antibodies, SA, oligonicleotides for HCR, and redox probes labelled SA on Au/SiO_2_-Fe_3_O_4_ NPs. The sensor exhibited high sensitivity for the simultaneous detection of the four biomarkers (Table 3). 

The human epidermal growth factor receptors ErbB2, HER2, and HER3 and carbohydrate antigen 15-3 (CA 15-3) are biomarkers of breast cancer. Ali et al. [106] constructed a microfluidic ErbB2 immunosensor based on porous GF electrode modified with electrospun carbon-doped TiO_2_ nanofibers and ErbB2 antibodies. Tabasi et al. [24] developed a HER2 specific aptamer-based HER2 immunosensor by depositing rGO-CS film on GCE and immobilizing amino-terminated aptamers on this film. Methylene blue (MB) was used to probe biointerface events. Rajesh et al. [107] fabricated a sensitive HER3 biosensor, what is based on graphene FET decorated with antibody-functionalized PtNPs. PtNPs were attached to graphene using the bifunctional 1-methyl pyrene amine linker, and thiol-containing single-chain variable fragment antibodies (scFv) were immobilized on PtNPs. Akter et al. [108] constructed a sandwich-type CA 15-3 immunosensor using GO/1-pyrenecarboxylic acid (Py-COOH) as sensor probe and MWCNT-supported ferritin as labels. GO/Py-COOH was deposited on cysteamine (Cys) self-assembled monolayer (SAM) modified gold electrode, and anti-CA 15-3 antibodies were immobilized on both GO/Py-COOH and MWCNT/ferritin labels.

**Figure 5:**
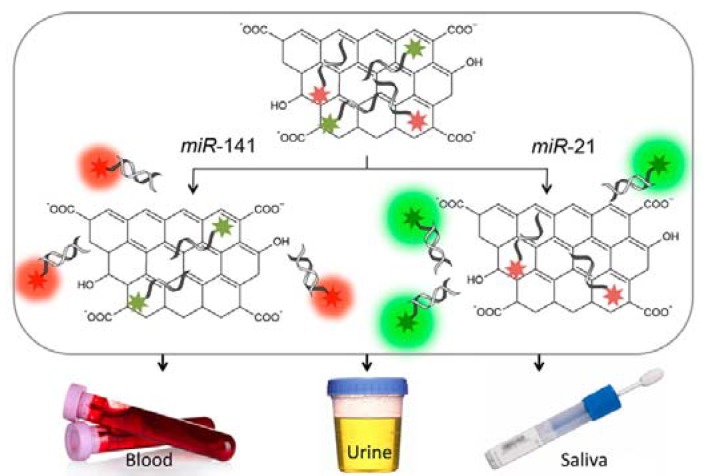
Illustration of the nGO/FAM-anti-miRNA-21/Cy5-anti-miRNA-141 assembly of the two-colour GO based fluorescence immunosensor. The surface adsorbed probe strands hybridize to the complementary target microRNA (miR) resulting in recovery of the fluorescence, whereas the nontarget miR does not change the fluorescence due to an absence of hybridization [115]. Copyright 2014. Reproduced with permission from American Chemical Society.

Mesothelin (MSLN) antigen is a biomarker for ovarian and pancreatic cancer. A sandwich-type MSLN immunosensor was fabricated by Shiddiky et al. [109] based on high-density poly(*N*-isopropyl acrylamide) (pNiPAM) antifouling brush modified ITO electrode and an electroactive label. Polyclonal MSLN antibodies were immobilized on the terminus of the pNiPAM brushes using alkyne-azide ‘click’ reaction. The bioconjugate label was prepared by immobilizing amine-functionalized CdSe QDs and SA on GO nanosheets, and linking single-chain variable antibody fragments (scFv) of MSLN comprising only the antigen recognition region on CdSe-QD/GO labels utilizing the streptavidin-biotin interaction. The tumor-associated glycoprotein TAG-72 (cancer antigen 72-4, CA72-4) is a gastric cancer biomarker. Wu et al. [110] constructed a CA72-4 immunosensor by modifying GCE with rGO-tetraethylenepentamine (rGO-TEPA) for effective immobilization of primary anti-CA72-4 antibodies, and adsorbing secondary anti-CA72-4 antibodies onto dumbbell-like PtPd-Fe_3_O_4_ NPs to prepare labels. Tumor necrosis factor-alpha antigen (TNF-α) is a recognized tumor marker and correlated with various diseases. Mazloum-Ardakani and Hosseinzadeh [111] developed an enzyme-free TNF-α aptasensor based on Ag@Pt core-shell NPs functionalized rGO nanosheets (Ag@Pt-rGOs) as labels and AuNPs functionalized rGO/chitosan (Au-rGO/CS) nanocomposite modified SPCE as sensing platform. Thiolated TNF-α aptamers were immobilized on both Au-rGO/CS and Ag@Pt NPs to capture TNF-α. Distinct miRNA expression patterns are associated with various tumor types. Cheng et al. [112] developed a miRNA-21 biosensor applying thiolated ssDNA (DNA1) and a biotin-labelled reporter ssDNA (DNA2) as target capturers and streptavidin-modified, Cd^2+^ functionalized titanium phosphate nanospheres (TiP-Cd^2+^) as labels. PEI-modified rGO/AuNP composites were drop-casted on GCE and DNA1 was immobilized on AuNP via chemisorptions. TiP-Cd^2+^ was attached to DNA2 via biotin-avidin conjugation as signal tag. Tu et al. [113] constructed a miRNA-126 fluorescence sensor based on GO fluorescence quenching and site-specific DNA cleavage of *Rsa*I endonuclease. The assay is based on the fluorescence recovery of the 66-base FAM-labelled probe ssDNA assembled on GO, after hybridization of ssNDA with miRNA-126 and cleavage of the dsDNA by *Rsa*I. Cai et al. [114] developed a miRNA let-7b biosensor based on AuNPs decorated graphene FET. The sensor was fabricated by drop-casting rGO onto the sensing channel of FET, depositing AuNPs onto the surface of rGO, and immobilizing target complementary peptide nucleic acid (PNA) probe on AuNPs. Hizir et al. [115] constructed a two-colour fluorescence sensor for the simultaneous detection of prostate cancer miRNA-21 and miRNA-141 biomarkers in body liquids. The sensor is based on FAM-labelled anti-miR-21 and Cy5-labeled anti-miR-141 ssDNA. The working principle of the sensor is shown in Figure 5. 

Thrombin is a biomarker of pulmonary metastases. A label free optical sensing platform was constructed by Li et al. [116] for thrombin detection, what was based on [Ru(2,2′-bipyridine)_2_(2-(2-methoxylphenyl)-imidazo[4,5-f][1,10]phenanthroline)]^2+^ (RuOMO), GO, and a thrombin specific aptamer pair. Thrombin detection was achieved by restoration of the fluorescence of RuOMO pre-quenched by GO. The cyclin A_2_ protein is a prognostic indicator in early-stage cancers. A fluorescence and an electrochemical cyclin A_2_ biosensor were developed by Wang et al. [117] and Feng et al. [118], respectively. The optical sensor was based on GO as the fluorescence quencher and on a fluorescent-labelled FITC-HAKRRLIF peptide as target recognition element [117]. The electrochemical sensor was constructed by modifying GCE with meso-tetra(4-carboxyphenyl)porphyrin (TCPP) modified chemically converted graphene (CCG) and immobilizing hexapeptide RWIMYF on the surface as specific binding site for cyclin A_2_ [118]. This latter sensor exhibited higher sensitivity with LOD of 0.32 pM. 8-hydroxy-2′-deoxyguanosine (8OHdG) is regarded as a cancer risk biomarker and indicative of a number of other disorders, such as cardiovascular and neurodegenerative diseases, and diabetes. Shahzad et al. [119] and Jia et al. [120] fabricated 8OHdG biosensors by modifying GCE with S-doped rGO (SRGO) and ss-DNA functionalized GR nanosheets, respectively. Sensors exhibited excellent electrocatalytic activity toward the oxidation of 8OhdG and comparable LOD and detection range (Table 3). Monitoring l-lactate concentration is important for early diagnosis and treatment of cancer, because tumor metabolism releases a high amount of lactate into the extracellular space. Azzouzi et al. [121] developed a l-lactate biosensor by drop casting a mixture of rGO-AuNP nanocomposite and L-lactate dehydrogenase (LDH) in a sol gel matrix onto SPCE. Folic acid protein (FAP) is a biomarker of many human epithelial-derived tumors. He et al. [122] constructed a FAP biosensor based on the strong binding affinity of FAP to folic acid (FA). The working electrode was prepared by electrophoretic deposition of rGO onto a gold electrode and functionalizing rGO with FA. The overexpression of matrix metalloproteinases (MMPs) is related to tumor invasion and metastasis. Song et al. [123] developed a fluorescence turn-on sensor for MMP-2 detection by linking amino-terminated fluorescein isothiocyanate-labelled peptide (Pep-FITC) to GO. The pre-quenched fluorescence of the FITC was restored upon contact with MMP-2, because MMP-2 selectively cleaved the peptide and FITC was released from the GO surface. The cytokeratin-19 fragment CYFRA-21-1 is a biomarker of oral cancer. Kumar et al. [124] constructed a biosensor based on zirconia decorated rGO to detect CYFRA-21-1 in saliva. The sensor was fabricated by electrophoretic deposition of 3-aminopropyl triethoxy saline (APTES) functionalized nanostructured ZrO_2_ decorated rGO onto ITO electrode and immobilizing anti-CYFRA-21-1 antibodies on the thin nanocomposite film. Squamous cell carcinoma antigen (SCCA) is a tumor marker for cervical cancer. Wu et al. [22] and Gao et al. [125] developed sandwich-type immunosensors for SCCA detection based on N-doped graphene (N-GS)–chitosan composite film and N-GS film on GCE, respectively, using anti-SCCA. Pt-Fe_3_O_4_ and Pd-Au/carbon NPs were used as trace labels, respectively, and the latter sensor exhibited better sensor performance with a LOD of 1.7 pg/mL [125]. The human tissue polypeptide antigen (hTPA) is a universal tumor marker. Wang et al. [126] constructed a sandwich-type hTPA immunosensor by immobilizing primary and secondary hTPA antibodies on GO modified GCE and Pd-Pt bimetallic nanocrystals, respectively. The tumor specific growth factor (TSGF) is a malignant tumor biomarker. A sandwich-type TSGF immunosensor was developed by Yu et al. [127] by modifying a GCE with rGO-TEPA and primary TSGF antibodies. Sensor labels were fabricated by anchoring secondary antibodies on Ag@CeO_2_ NP nanocomposite. The nuclear matrix protein 22 (NMP22) is a biomarker for bladder cancer. A highly sensitive NMP22 immunosensor was developed by Ma et al. [128] by immobilizing NMP22 antibodies on trimetallic AuPdPt NPs and depositing these bioconjugates on rGO-TEPA modified GCE. Apolipoprotein A II protein (APOA2) is also a bladder cancer biomarker and, similarly to NMP22, can be detected in urine. Chen et al. [129] developed an APOA2 biosensor based on polycrystalline silicon nanowire field-effect transistor (poly-SiNW-FET) and magnetic graphene (Fe_3_O_4_ NPs-rGO) with long-chain acid groups (MGLA). Anti-APOA2 antibodies were immobilized on MGLA and this bioconjugate was immobilized on poly-SiNW-FET. 

#### 3.2.2. Graphene-Based Biosensors for Disease Biomarker Detection

The amyloid beta (Aβ) peptide and mRNA BACE-1 are biomarkers of Alzheimer’s disease. Kim et al. [130] developed a sensitive chemiresistor-type wafer-scale rGO biosensor for Aβ_40_ detection. Aβ antibodies were immobilized on rGO for target capture. Vilela et al. [96] constructed a BACE-1 optical fluorescence turn-on biosensor based on BACE-1 complementary capture ssDNAs immobilized on NaYF4:Yb,Er upconversion NPs (UCNPs) as emitters and GO as the fluorescence quencher (Table 4). 

Cardiac troponin-I (cTnI) is a biomarker of acute myocardial infarction. A sandwich-type cTnI biosensor was constructed by Liu et al. [131], what was based on GO-Ph-AuNP modified GCE as sensing platform and GO tailored with ferrocene (FcGO) as signal reporter labels. cTnI capture antibodies were immobilized on both the working electrode surface and FcGO labels using aryldiazonium salt coupling chemistry. 3,3′,5-triiodothyronine (T3) is a widely used diagnostic marker of thyroid disease. Liao et al. [132] constructed a T3 biosensor based on modified GCE working electrode and GO bioconjugate labels. The sensing platform of the sensor was prepared by electropolymerizing l-lysine (LL) on GCE, electrodepositing AuNPs, and immobilizing T3 antibodies on the modified electrode surface. Labels were prepared by co-immobilizing Ru(bpy)_3_^2+^ and T3 antibodies on Fe_3_O_4_ loaded GO nanosheets. Procalcitonin (PCT) is a diagnostic biomarker of septicemia disease. Liu et al. [133] developed a sandwich electrochemical strategy for PCT detection. The working electrode of the sensor was constructed by electrodepositing rGO-Au nanocomposite film on GCE and immobilizing PCT antibodies on this film. Bioconjugate labels were prepared by binding anti-PCT to thionine (Thi) and linking this complex to single-walled carbon nanohorns (SWCNHs)/hollow Pt chains (HPtCs) together with HRP for dual synergy amplification. Estriol is one of the estrogens produced in women and abnormal esteriol level is associated with various diseases. Kushwaha et al. [134] designed a fluorescent estriol sensor based on fluorescence enhancement of GO upon bonding of estriol, due to radiative energy transfer. 

d-amino acids are biomarkers of various diseases, because normally only l-amino acids are involved in physiological processes. d-Tyrosine (Tyr), for example, is a renal biomarker. Martín et al. [135] constructed a biosensor for the analysis of D and L tyrosine and methionine by casting reduced GO nanoribbons (RGONR) onto SPCE. The sensing strategy was based on a dual electrochemical and enzymatic approach involving d-amino acid oxidase (DAAO) for d-amino acid sensing and direct electrochemical sensing for the l-enantiomer. Abnormalities in insulin secretion and activity lead to various types of diabetes and increased risk factors of various diseases. Yagati et al. [23] constructed an insulin sensor based on silver nanoflower (AgNF) decorated rGO modified micro-disk electrode arrays and anti-insulin antibodies anchored on the working electrode (Table 4). 

Dopamine (DA) and uric acid (UA) are important biomarkers of various diseases; therefore several sensor platforms were constructed for their detection. Sun et al. [136] fabricated a DA and UA biosensor by drop-casting short GO nanoribbons (GONR) and Nafion suspension onto GCE. A sensitive biosensor based on ZnO nanowire arrays/graphene foam (GF) composite modified ITO electrode was developed by Yue et al. [137] for the selective detection of UA and DA in serum (Table 4). A DA sensor based on 3D-rGO modified GCE was fabricated by Yu et al. [138]. The sensor exhibited an excellent sensing performance and high selectivity. Liu et al. [139] constructed a DA sensor based on assembled multilayer of polyamidoamine (PAMAM) dendrimer stabilized AuNPs and polysodium 4-styrenesulfonate (PSS) functionalized rGO on GCE. The sensor exhibited excellent sensing performance with a small LOD of 20 nM. Bai et al. [140] fabricated a DA sensor based on a GCE modified by three layers of highly reduced GO film prepared by self-assembly from rGO dispersion at the DMF-air interface through evaporation-induced water-assisted thin film formation. The three rGO layers modified electrode exhibited higher activity to dopamine than a single layer modified electrode.

Increased lactate level in serum is an indicator of several diseases. Manna and Raj [141] developed a sensor to detect l-lactate by depositing *p*-nitrophenyl functionalized rGO on GCE, generating a surface confined redox mediator (rGO-PhNHOH), and immobilizing l-lactate dehydrogenase (LDH) on the electrode surface. The glucose detection in body fluids is essential for monitoring diabetes. Glucose sensors, in general, are based on the immobilization of glucose oxidase (GlOx) on top of modified electrode surfaces or on incorporation of GlOx into the modifying thin layer of the bare electrode. Assembled graphene sheets provide a possibility to incorporate GlOx in between graphene layers and to control layer thickness for improving sensor performances. Liu et al. [142] fabricated a glucose sensor by self-assembling GlOx and CS functionalized graphene platelets on GCE by electrostatic attraction. A glucose biosensor was fabricated by Barsan et al. [143] by self-assembling positively charged CS containing GlOx and nitrogen doped graphene (NGR) with negatively charged poly(styrene sulfonate) (PSS) on gold electrode. Yan et al. [144] constructed a glucose sensor by assembling poly(diallyldimethylammonium chloride) protected Prussian blue NPs (PDDA-PB), GlOx, and rGO on GCE. Zeng et al. [145] prepared a multilayer film on GCE by alternating deposition of poly(ethyleneimine) (PEI), pyrene-grafted poly(acrylic acid) modified rGO (PAA-rGO), and GlOx. Gu et al. [146] prepared a multilayer on GCE by self-assembling amine-terminated ionic liquid (IL) modified rGO and sulfonic acid (SA) functionalized rGO sheets, followed by immobilizing GlOx on the top layer. This latter sensor exhibited superior performance with LOD of 3.33 µM (Table 4). Graphene aerogel (GA) possesses a much higher electrical conductivity than casted graphene powder, because constituent graphene sheets are chemically bonded. A glucose sensor exhibiting a LOD of 0.597 µM was fabricated by Wang et al. [147] by casting GA/AuNPs onto GCE and immobilizing GlOx in the aerogel framework. Electrospun fiber membranes often show superior performance in sensor construction compared to casted film membranes due to very high porosity and large surface area. Su et al. [148] fabricated a glucose sensor by electrospinning a mixture of poly(vinyl alcohol) (PVA), CS, GO, and GlOx directly onto a Pt electrode and depositing a thin layer of Nafion onto the modified electrode surface for anti-interference effects. Liang et al. [149] used single graphene coated silk fibers for sensor construction. A composite film of these fibers was prepared by vacuum filtration of a mixed solution of GO and silk fibers, followed by chemical reduction with ascorbic acid. Pt nanospheres were electrochemically deposited onto the film surface and GlOx was immobilized on the electrode by cross-linking to pre-deposited BSA. Zhang et al. [150] constructed a very sensitive glucose sensor with a LOD of 1 nM by casting graphene decorated MnCo_2_O_4_ composite nanofibers onto GCE obtained by calcinating electrospun rGO/Mn(Ac)_2_/Co(Ac)_2_/polyvinyl pyrrolidone (PVP) composite fibers at high temperature.

### 3.3. Carbon Quantum-Dot-Based Biomarker Biosensors

The application of carbon quantum-dots in the construction of biomaker sensors is currently in its initial stage [17]. Graphene QDs were used to date to construct electrochemiluminescence (ECL) and fluorescence biosensors (Table 5). 

A label-free ECL immunosensor was developed by Wu et al. [151] for PSA quantification by depositing Au/Ag-rGO modified with aminated GR-QDs and GO-QDs onto GCE and immobilizing anti-PSA on the sensor surface. Dong et al. [152] constructed a label-free biosensor for CEA detection by modifying a GCE with rGO-QD-AuNP nanohybrids and immobilizing anti-CEA on the electrode surface. An improved CEA sensor reaching a LOD of 0.6 pg/mL was developed by Li et al. [153] using nanoporous gold/chitosan modified paper working electrode (PWE) as sensor platform and GO-QD functionalized Au@Pt core-shell NPs as signal labels, as well as anti-CEA antibodies immobilized on both the sensor surface and labels. The LOD of this sensor was comparable to those of best performing CNT and graphene based electrochemical sensors. A sensitive ECL immunosensor was fabricated by Yang et al. [154] for the detection of carbohydrate antigen 199 (CA199). The sensor platform was prepared by modifying GCE with Au and Ag NPs-modified rGO, and immobilizing anti-CA199 on the sensor surface. Signal amplification was based on anti-CA199 immobilized on GO-QD modified PtPd-nanochains. 

**Figure 6:**
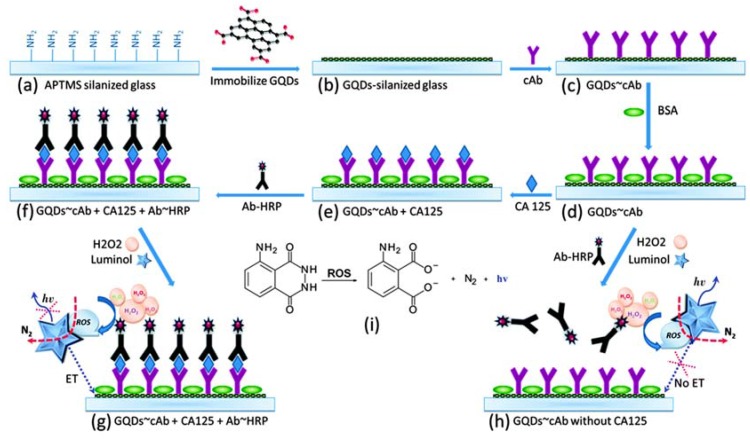
Illustration of the assembly of the carcinoma antigen-125 (CA125) immunoassay and the detection principle (Ab = antibody, APTMS = 3-aminopropyl-trimethoxysilane, GQD = graphene quantum-dot, HRP = horseradish peroxidase, ROS = reactive oxigen species) [156]. Copyright 2014. Reproduced with permission from Royal Society of Chemistry.

Zhao et al. [155] constructed a fluoroimmunoassay based on the regulation of the resonance energy transfer (RET) between rGO acceptors and GO-QD donors for human immunoglobulin G (IgG) detection. Anti-IgG antibodies were immobilized on GO-QDs, what adsorbed on rGO quencher, but released from rGO upon interaction with target IgGs. Al-Ogaidi et al. [156] developed a sensitive biosensor for the detection of the ovarian cancer biomarker CA125 utilizing the chemiluminescence RET to graphene QDs. QDs were immobilized on glass chips and anti-CA125 antibodies were linked to QDs through amide conjugation. Horseradish peroxidase (HRP)-labelled anti-CA125 antibodies were used as signal labels (see Figure 6). 

## 4. Conclusions and Outlook

The development of devices for early detection of diseases and treatment monitoring is of vital importance for clinical diagnosis and therapy. Biomarkers of diseases provide valuable information, however, their sensitive and reliable point-of-care measurement is challenging due to their low concentration in complex biological media. The sensitivity of electrochemical and optical biosensors now reached the limit of detecting biomarkers in body fluids in very low concentration, what provides a route for the routine application of non- or minimally invasive sampling techniques based on blood, urine, saliva, tear, and sweat. Thus biosensors have the potential to provide cheap, fast, highly accurate, sensitive, and reproducible results, and require only minimal technical expertise and system maintenance. These sensors have shown to be overcome obstacles of classical techniques involving invasive biopsies, expensive and complex labelling processes, and time-consuming analysis. A wide variety of sensing techniques are used currently for biomarker detection. It is difficult, however, to recommend which detection method is the most appropriate. The selection is not obvious, taking into account many parameters including the analyte, sample matrix, assay format, labelling requirements, miniaturizations and cost. Since electrode material and architecture play critical roles in sensor performances, great efforts have been done to develop new sensor platforms by introducing new materials. Carbon nanotubes and graphenes became essential elements of sensor platforms during the last decade due to their excellent electrical, mechanical, and optical properties, and it is expected that novel functionalization will expand their application possibilities. Carbon quantum dots are promising new platform elements and more attention is expected for these nanomaterials in the future. There has been a quest for detection limit in the past, but facile and economical considerations for sensor designs are getting more and more important. Most of sensor developments thus far have considered the detection of a single target. However, simultaneous measurement of multiple biomarkers can improve the diagnostic value, because many of disease markers are indicative of multiple diseases. Development of multi-analyte immunosensors are still in its early stage, and future researches are expected to move into this direction. The antibody-antigen interaction is very specific and this is the method of choice currently for recognition element designs of biosensors. Antibodies, however, are expensive and efforts are made to replace antibodies with cheaper and more stable recognition elements, such as aptamers, engineered single-chain variable antibody fragments, and organic molecules.

There is a large number of biosensors developed in recent years that are able to provide sensitivity and selectivity requirements related for practical applications, however, this is not enough for translation into industrial production and commercialization. Complicated sensor assembly processes, expensive materials, possible undesired properties at nanoscale and lack of storage stability are some limiting factors that preventing their mass production. Methods for producing identical sensor batches and scaling-up to mass production, as well as integration of biosensors into automated and miniaturized systems are yet to be developed. In addition, many of currently developed biosensors are not validated using large numbers of patient samples. Although carbon based biosensors research for cancer and disease detection is currently still at advanced laboratory stage, it has already provided a promise and vision about future disease diagnosis and health monitoring. Further progress in this field is expected to lead to development of biomarker sensors for routine clinical applications. Therefore, based on current progress, there is a bright future for carbon nanomaterial-based biosensors and their development along with involvement of new biomarkers will continue.

## Figures and Tables

**Table 1:** CNT(Carbon nanotube)-based cancer biosensors.

Sensor Platform//Label	Analyte	Instr. Techn.^a^	Linearity Range	LOD	References
Pt/SWCNT/anti-PSA	PSA	CHI	n.a.	0.25 ng/mL	[33]
GCE/starch-MWCNT-Nafion/AuNP/anti-PSA	PSA	CV	0.01–0.5, 0.5–3.0 ng/mL	7 pg/mL	[34]
PG/SWCNT/anti-PSA//anti-PSA/MWCNT/HRP	PSA	AMP	0.4–40 ng/mL	4 pg/mL	[35]
GCE/PPy@MWCNT/AuNP/anti-PSA//HRP-anti-PSA	PSA	DPV	0.002–20 ng/mL	1 pg/mL	[36]
FET/CNT/anti-OPN	OPN	CHI	1 pg/mL–1 µg/mL	1 pg/mL	[37]
Au/ITO/SWCNT/anti-OPN	OPN	CHI	1 pg/mL–1 µg/mL	0.3 pg/mL	[38]
Au/SWCNT/NDI/SWCNT/AuNP/TSP-cDNA//AuNP-hemin-G-quadruplex DNAzyme	miRNA-21	DPV	10 fM–1 nM	1.95 fM	[39]
GCE/ox-MWCNT/poly(JUG-JUGA)/cDNA	miRNA-141	SWV	10 fM–100 pM	8 fM	[40]
GSPE/rGO/ox-MWCNT/cDNA//anti-miRNA/HRP-anti-miRNA	miRNA-141	SWV	10 fM–1 nM	10 fM	[41]
Au/SWCNT/protein p19	miRNA-122a	CHI	1 aM–10 fM	1 aM	[42]
GCE/CNT/NH_2_-HP DNA/CP/RCA	miRNA let-7	DPV	10–1000 fM	1.2 fM	[43]
GCE/MWCNT/cDNA	miRNA-24	DPV	1 pM–1 nM, 1–10 nM	1 pM	[44]
Au/CS-MWCNT-Thi/anti-CEA	CEA	DPV	1 pg/mL–100 ng/mL	0.5 pg/mL	[45]
CP/PEDOT:PSS-CNT/anti-CEA	CEA	AMP	2–15 ng/mL	n.a.	[46]
GCE/(PB-NP/RGO-MWCNT)_5_/AuNP/anti-CEA	CEA	DPV	0.2–1, 1–40 ng/mL	60 pg/mL	[47]
GCE/(PB-NP/MWCNT-PEI-AuNP)_5_/CS-AuNP/anti-CEA	CEA	AMP	0.5–2, 2–160 ng/mL	80 pg/mL	[48]
GCE/(PDDA-MWCNT/PSS)_2_/PDDA-MWCNT/AuNP/anti-CEA	CEA	AMP	0.1–2, 2–160 ng/mL	60 pg/mL	[49]
Au/l-Cys/anti-CEA//MWCNT/PDDA/HRP/ConA/HRP-anti-CEA	CEA	DPV	0.05–200 ng/mL	18 pg/mL	[50]
SPCE/MWCNT/anti-CEA//AuNR/GlOx/anti-CEA	CEA	DPV	0.01–100 ng/mL	4.2 pg/mL	[51]
GCE/RGO-MWCNT/Pt/anti-CEA//Pt/Fe@CD/anti-CEA	CEA	ECL	0.003–600 ng/mL	0.8 pg/mL	[52]
ITO/MWCNT-PABA/anti-CEA//MWCNT-CR/C_60_/anti-CEA	CEA	PEC	0.001–100 ng/mL	0.1 pg/mL	[53]
GCE/GS-NH_2_/anti-CEA//PdPt/MWCNT-NH_2_/anti-CEA	CEA	AMP	0.001–20 ng/mL	0.2 pg/mL	[54]
GCE/MWCNT-ZnO/anti-CA125	CA125	DPV	0.001–1000 U/mL	0.00113 U/mL	[55]
GCE/MWCNT-Nafion/Co(bpy)_3_^3+^/AuNP/anti-CA125	CA125	CV	1–30, 30–150 U/mL	0.36 U/mL	[56]
QCM/PLL/HA/MWCNT/anti-CA 19-9	CA 19-9	QCM	12.5–270 U/mL	8.3 U/mL	[29]
Ta/MWCNT/AuNP/cDNA	TP53	EIS	1 fM–100 nM	10 aM	[57]
GCE/MWCNT-PA6-PTH/anti-AG_p53_//HRP@anti-AG_p53_	AG_p53_	DPV	2–2000 pg/mL	1 pg/mL	[58]
GCE/MWCNT-PA6/PTH/ssDNA1//AuNP-ssDNA2	K-ras	DPV	0.1–100 pM	30 fM	[59]
FET/SWCNT-galactose	Galectin-3	CHI	156–312.5 ng/mL	n.a.	[60]
Au/PoPD/AuNP/aptamer//aptamer/Thi/AuNP/SiO_2_@MWCNT	MUC 1	DPV	1–100 nM	1 pM	[61]
MWCNT-CILE/AuNP/HDT/AuNP/anti-HER2	HER2	EIS	10–110 ng/mL	7.4 ng/mL	[62]
SPCE/SWCNT/anti-HER-3	HER-3	EIS	2–14 fg/mL	2 fg/mL	[63]
PG/SWCNT/anti-MMP-3//anti-MMP-3/polystyrene/HRP	MMP-3	AMP	4–300 pg/mL	4 pg/mL	[64]
GCE/CS-AuNP/anti-AFP//HOOC-SWCNT-MnO_2_	AFP	LSV	0.2–100 ng/mL	40 pg/mL	[65]
SPCE/SWCNT/WGA	AFP	EIS	1–100 pg/mL	0.1 pg/mL	[66]
GCE/AuNP/anti-AFP//Pb^2+^@Au@MWCNT-Fe_3_O_4_/anti-AFP	AFP	AMP	10 fg/mL–100 ng/mL	3.33 fg/mL	[67]
GRT/SWCNT-CS/anti-anti-MAGE A2 or A11	anti-MAGE A2	DPV	5 fg/mL–50 ng/mL	n.a.	[68]
	anti-MAGE A11				
SPCE/HOOC-Phe-DWCNT/Mix&Go/anti-TNF,anti-IL/ /biotin-anti-TNF, biotin-anti-IL/poly-HRP-strept	TNF-α	AMP	1–200 pg/mL	0.85 pg/mL	[69]
	IL-1β		0.5–100 pg/mL	0.38 pg/mL	

^a^ Instrumental techniques: AMP = amperometry, EIS = electron impedance spectroscopy, FS = fluorescence sensor, CHI = chemiresistor, CHA = chronoamperometry, CL = chemiluminescence, CV = cyclic voltammetry, DPASV = differential pulse anodic stripping voltammetry, DPV = differential pulse voltammetry, ECL = electrochemiluminescence, LSV = linear sweep voltammetry, PEC = photoelectrochemical, PT = potentiometry, QCM = quartz crystal microbalance, SWV = square wave voltammetry, SWASV = square wave anodic stripping voltammetry, n.a. = not available.

**Table 2:** CNT-based disease biosensors.

Sensor Platform//Label	Analyte	Instr. Techn.^a^	Linearity Range	LOD	References
SPE-MWCNT//anti-Mgb	myoglobin	EIS	0.1–90 ng/mL	0.08 ng/mL	[70]
GCE/c-MWCNT-CS/Thi/AuNP/anti-netrin 1	netrin 1	DPV	0.09–1800 pg/mL	0.03 pg/mL	[71]
SPCE/MP-anti-MPO/SWCNT	MPO	CHA	n.a.	55 ng/mL	[72]
GCE/DMF-MWCNT-EMIMBF_4_/CS-CeO_2_NP/anti-MPO	MPO	CV	5–300 ng/mL	0.2 ng/mL	[73]
ITO/PoPD-MWCNT-EMIMBr/AuNP/anti-MPO	MPO	CV	0.2–23 and 23–300 ng/mL	0.05 ng/mL	[74]
GCE/Nafion-ZnS-c-MWCNT-ChOx	cholesterol	CV	1.3–11.6 mM	0.26 mM	[75]
FET/CNT/loop peptide	ab-AQP4	CHI	n.a.	1 pg/mL	[76]
FET/SWCNT/anti-ag-Lyme	ag-Lyme	CHI	1–3000 ng/mL	1 ng/mL	[77]
SPCE/PTCA-MWCNT/AAT aptamer//ALP-anti-AAT/AgNP	AAT	DPV	0.05–20 pM	0.01 pM	[78]
FET/SWCNT/Au/anti-amyloid-β	amyloid-β	CHI	1 pg/mL–1 ng/mL	1 pg/mL	[79]
GCE/c-MWCNT/ZnO_2_-NP/AChE-ChlO	choline	CV	0.05–200 µM	0.01 µM	[80]
GCE/PAMAM-AuNP-MWCNT/anti-SirT1//SiO_2_@Au NP/HRP-anti-SirT1	SirT1	DPV	20 pg/mL–500 ng/mL	12.5 pg/mL	[81]
Au/c-MWCNT-PS/CFFCP1 peptide//anti-ACPA-HRP	ACPA	AMP	n.a.	n.a.	[82]
Au/SWCNT/CSPR peptide//anti-ACPA	ACPA	QCM	n.a.	n.a.	[83]
GCE/MWCNT/PB/CS/ anti-Tcd B//GO/HRP-anti-Tcd B/HRP	Tcd B	DPV	0.003–320 ng/mL	0.7 pg/mL	[84]
Cu/MWCNTs-MIP	dopamine	PT	1–10,000 nM	1.0 nM	[85]
CCE/MWCNTs-MIP	dopamine	DPASV	0.75–34 ng/mL	0.21 ng/mL	[86]
Au/ssDNA-SWCNT/PABA/Nafion	dopamine	DPV	n.a.	40 pM	[87]
GCE/c-MWCNT/SiO_2_	dopamine	DPV	0.5–6 µM	14 nM	[88]
GCE/ox-MWCNT/SiO_2_	uric acid	DPV	0.5–10 µM	0.068 µM	[88]
GCE/SWCNT/Thi/UOx	uric acid	AMP	2 µM–2 mM	0.5 µM	[89]
GCE/PPy/c-MWCNT/GlOx	glucose	AMP	4–50 mM	0.2 mM	[90]
Au/SWCNT/PPy/GlOx	glucose	AMP	0.56–100 mM	0.05 mM	[91]

^a^ Instrumental techniques: see Table 1 for abbreviations.

**Table 3:** Graphene-based cancer biomarker biosensors.

Sensor Platform//Label	Analyte	Instr. Techn.^a^	Linearity Range	LOD	References
GO/UCNP/ssDNA	PCA3	FS	n.a.	0.5 pM	[96]
FET/(PDDA+PSS)_2_(PDDA+GR)_5_/anti-PSA	PSA	CHI	4 fg/mL–4 µg/mL	0.11 fM	[97]
FET/(PDDA+PSS)_2_(PDDA+GR)_5_/anti-PSA	PSA	CHI	0.4 fg/mL–4 µg/mL	11 aM	[98]
FET/GR/anti-ANXA2, or anti-ENO1, or anti-VEGF	ANXA2, ENO1, VEGF	CHI	1 pg/mL–1 µg/mL	0.1 pg/mL	[99]
FET/(PDDA+GR)_2_(PDDA+TiO_2_) (PDDA+GR)_2_/anti-ANXA2, or anti-ENO1, or anti-VEGF	ANXA2, ENO1, VEGF	CHI	100 fg/mL–1 µg/mL	100 fg/mL	[100]
GF/pDA/ConA/HRP-anti-CEA	CEA	DPV	0.1–750 ng/mL	90 pg/mL	[25]
GCE/GR/SA-CS//HP-DNA-AuNR-HRP	CEA	DPV	5 pg/mL–50 ng/mL	1.5 pg/mL	[26]
PWE/AuNPs/PANI/anti-CEA,anti-AFP//rGO/MB,Fc-COOH/anti-CEA,anti-AFP	CEA, AFP	DPV	1 pg/mL–100 ng/mL	0.5 pg/mL, 0.8 pg/mL	[101]
GCE/CS-AuNP/anti-CEA,anti-AFP//anti-CEA-TB-CGS/anti-AFP-PB-CGS	CEA, AFP	DPV	0.5–60 ng/mL	0.1 ng/Ml, 0.05 ng/mL	[102]
GCE/IL-rGO/anti-CEA,anti-AFP//anti-CEA-PtNP-Cd^2+^,anti-AFP-PtNP-Cu^2+^	CEA, AFP	DPV	0.05–200 ng/mL	0.002 ng/mL, 0.05 ng/mL	[103]
GCE/IL-rGO/PSS/anti-CEA,anti-AFP,anti-PSA//anti-CEA-Thi-CAuNP,anti-AFP-Cd^2+^-CAuNP,anti-PSA-DAP-CAuNP	CEA, AFP, PSA	SWV	0.01–100 ng/mL	2.7 pg/mL, 3.1 pg/mL, 4.8 pg/mL	[104]
GCE/GR-AuNP/anti-CEA,AFP,CA125,PSA//SA/biotin-dsDNA/SA/biotin-anti-CEA,AFP,CA125,PSA/Au/SiO_2_-Fe_3_O_4_	CEA, AFP, CA125 PSA	DPV	0.2–600, 0.2–800, 0.2–1000, 0.2–800 pg/mL	48 fg/mL, 62 fg/mL, 77 fg/mL, 60 fg/mL	[105]
GF-nTiO_2_/anti-ErbB2	ErbB2	DPV	1.0 fM–0.1 µM	n.a.	[106]
GCE/rGO-CS/aptamer/MB	HER2	DPV	0.5–2, 2–75 ng/mL	0.21 ng/mL	[24]
FET/GR/PtNP/scFv-anti-HER3	HER3	CHI	300 fg/mL–300 ng/mL	300 fg/mL	[107]
Au/Cys/GO/Py-COOH/anti-CA 15-3//anti-CA 15-3/MWCNT/Ferritin	CA 15-3	DPV	0.05–100 U/mL	0.009 U/mL	[108]
ITO/APES/pNiPAM/anti-MSLN//scFv-MSLN/GO/CdSe QDs	MSLN	SWASV	n.a.	0.5 pg/mL	[109]
GCE/rGO-TEPA/anti-CA72-4//anti-CA72-4/ PtPd-Fe_3_O_4_	CA72-4	EIS	0.001–10 U/mL	0.0003 U/mL	[110]
SPCE/AuNP-GR/CS/aptamer//aptamer-Ag@Pt-Gr	TNF-α	DPV	5–70 pg/mL	1.64 pg/mL	[111]
GCE/PEI-RGO/AuNP/cDNA1//cDNA2/TiP-Cd^2+^/Ru(NH_3_)_6_^3+^	miRNA-21	SWV	1 aM–10 pM	0.76 aM	[112]
GO/FAM-ssDNA	miRNA-126	FS	0.02–100 pM	3.0 fM	[113]
FET/rGO/AuNP/PNA	miRNA let7b	CHI	10 fM–100 pM	10 fM	[114]
GO/FAM-anti-miR-21/Cy5-anti-miR-141 ssDNA	miRNA-21 miRNA-141	FS	n.a.	2.0 nM, 1.2 nM	[115]
GO-RuOMO-aptamers	thrombin	FS	3.7–613 nM	0.76 nM	[116]
GO/ FITC-HAKRRLIF	cyclin A_2_	FS	n.a.	0.5 nM	[117]
GCE/CCG/TCPP/hexapeptide	cyclin A_2_	EIS	0.5–10 pM	0.32 pM	[118]
GCE/SRGO	8OHdG	DPV	2 nM–20 µM	1 nM	[119]
GCE/GR/ss-DNA	8OHdG	CV	0.0056–1.155, 1.155–11.655, 11.655–36.155 µM	0.875 nM	[120]
SPCE/RGO-AuNP/LDH	l-lactate	AMP	0.01–5 mM	0.13 µM	[121]
Au/rGO/FA	FAP	DPV	1–200 pM	1 pM	[122]
GO/ Pep-FITC	MMP-2	FS	10–150 ng/mL	2.5 ng/mL	[123]
ITO/rGO-ZrO_2_/APTES/anti-CYFRA-21-1	CYFRA-21-1	DPV	2–22 ng/mL	0.122 ng/mL	[124]
GCE/N-GS/CS/anti-SCCA//anti-SCCA/Pt-Fe_3_O_4_	SCCA	EIS	0.05–18 ng/mL	15.3 pg/mL	[22]
GCE/N-GS/anti-SCCA//anti-SCCA/Pd-Au/C	SCCA	EIS	0.005–2 ng/mL	1.7 pg/mL	[125]
GCE/GO/anti-hTPA//anti-hTPA/Pd-Pt NP	hTPA	EIS	0.005–15 ng/mL	1.2 pg/mL	[126]
GCE/rGO-TEPA/anti-TSGF//anti-TSGF/Ag@CeO_2_	TSGF	CV	0.5–100 pg/mL	0.2 pg/mL	[127]
GCE/rGO-TEPA/AuPdPt NP/anti-NMP22	NMP22	DPV	0.04–20 U/mL	0.01 U/mL	[128]
FET-poly-SiNW/MGLA-anti-APOA2	APOA2	CHI	19.5 pg/mL–1.95 µg/mL	6.7 pg/mL	[129]

^a^ Instrumental techniques: see Table 1 for abbreviations.

**Table 4:** Graphene-based disease biomarker biosensors.

Sensor Platform//Label	Analyte	Instr. Techn.^a^	Linearity Range	LOD	References
MEMS/rGO/anti-Aβ	Aβ_40_	CHI	100 fg/mL–100 pg/mL	100 fg/mL	[130]
GO/UCNP/ssDNA	BACE-1	FS	n.a.	0.5 pM	[96]
GCE/GO-Ph-AuNP/anti-cTnI//FcGO/anti-cTnI	cTnI	SWV	0.05–3 ng/mL	0.05 ng/mL	[131]
GCE/PLL/AuNP/anti-T3//Fe_3_O_4_@GO/Ru(bpy)_3_^2+^/anti-T3	T3	ECL	0.1 pg/mL–10 ng/mL	0.03 pg/mL	[132]
GCE/rGO-Au/anti-PCT//SWCNH/HPtC/Thi/anti-PCT/HRP	PCT	CV	1.0 pg/mL–2.0 ng/mL	0.43 pg/mL	[133]
GO	estriol	FS	1.3–10 nM	1.3 nM	[134]
SPCE/GONR/DAAO	d-Tyr	DPV	0.25–1.0 mM	60 µM	[135]
ITO/rGO-AgNF/MPA/anti-insulin	insulin	EIS	1–1000 ng/mL	70 pg/mL	[23]
GCE/GONR-Nafion	dopamine	DPV	0.1–8.5 µM	24 nM	[136]
GCE/GONR-Nafion	uric acid	DPV	0.1–8.5 µM	98 nM	[136]
ITO/GF/ZnO NWA	dopamine	DPV	0–40 µM	1 nM	[137]
ITO/GF/ZnO NWA	uric acid	DPV	0–40 µM	1 nM	[137]
GCE/3D-RGO	dopamine	DPV	5 µM–1 mM	0.17 µM	[138]
GCE/(PDDA)_1_(PSS-RGO/PAMAM-AuNPs)_20_	dopamine	DPV	1–60 µM	0.02 µM	[139]
GCE/(rGO)_3_(Nafion)	dopamine	LSV	0.5–30 µM	0.2 µM	[140]
GCE/rGO-PhNHOH/LDH	l-lactate	AMP	0–90 µM	2.5 µM	[141]
GCE/rGO-CS/GlOx	Glucose	CV	2–22 mM	20 µM	[142]
Au/[CS(NGR+GlOx)/ PSS/CS(NGR+GlOx]	Glucose	CHA	0.2–1.8 mM	64 µM	[143]
GCE/(rGO/PDDA-PB/GlOx/PDDA-PB)_3_	Glucose	AMP	0.1–6.5 mM	6 µM	[144]
GCE/(PEI/PAA-rGO)_3_(PEI/GlOx)_5_	Glucose	AMP	0 -10 mM	0.168 mM	[145]
GCE/(IL-RGO/SA-RGO)_5_/IL-RGO/GlOx	Glucose	AMP	10–500 µM	3.33 µM	[146]
GCE/GA-AuNP/GlOx	Glucose	AMP	50–450 µM	0.597 µM	[147]
Pt/GO/GlOx/CS/PVA fiber	Glucose	CV	5 µM–3.5 mM	5 µM	[148]
RGO/silk fiber/PtNP/GlOx	Glucose	AMP	10 µM–10 mM	1 µM	[149]
GCE/rGO/MnCo_2_O_4_ fiber/GlOx	Glucose	AMP	0.005–800 µM	1 nM	[150]

^a^ Instrumental techniques: see Table 1 for abbreviations.

**Table 5:** Quantum-dot-based biomarker biosensors.

Sensor Platform//Label	Analyte	Instr. Techn.^a^	Linearity Range	LOD	References
GCE/Au/Ag-rGO/GR-QD/GO-QD/anti-PSA	PSA	ECL	0.001–10 ng/mL	0.29 pg/mL	[151]
GCE/rGO-QD/AuNP/anti-CEA	CEA	ECL	0.02–80 ng/mL	10 pg/mL	[152]
PWE/Au/CS/anti-CEA/ /anti-CEA/GO-QD/Au@PtNP	CEA	ECL	0.001–10 ng/mL	0.6 pg/mL	[153]
GCE/PVP-rGO/AgNP/AuNP/anti-CA199//anti-CA199/GO-QD/PtPdNPs	CA199	ECL	0.002–70 U/mL	0.96 mU/mL	[154]
rGO/GO-QD/anti-IgG	IgG	FS	n.a.	10 ng/mL	[155]
GO-QD/anti-CA125/ /anti-CA125-HRP	CA125	CL	0.1–600 U/mL	0.05 U/mL	[156]

^a^ Instrumental techniques: see Table 1 for abbreviations.
